# Reliable Fast (20 Hz) Acquisition Rate by a TD fNIRS Device: Brain Resting-State Oscillation Studies

**DOI:** 10.3390/s23010196

**Published:** 2022-12-24

**Authors:** Rebecca Re, Ileana Pirovano, Davide Contini, Caterina Amendola, Letizia Contini, Lorenzo Frabasile, Pietro Levoni, Alessandro Torricelli, Lorenzo Spinelli

**Affiliations:** 1 Dipartimento di Fisica, Politecnico di Milano, Piazza Leonardo da Vinci, 32, 20133 Milan, Italy; 2 Istituto di Fotonica e Nanotecnologie, Consiglio Nazionale delle Ricerche, Piazza Leonardo da Vinci, 32, 20133 Milan, Italy; 3 Istituto di Tecnologie Biomediche, Consiglio Nazionale delle Ricerche, via Fratelli Cervi 93, 20090 Segrate, Italy

**Keywords:** time domain, functional near infrared spectroscopy, diffuse optics, brain, hemodynamics, resting-state brain oscillation

## Abstract

A high power setup for multichannel time-domain (TD) functional near infrared spectroscopy (fNIRS) measurements with high efficiency detection system was developed. It was fully characterized based on international performance assessment protocols for diffuse optics instruments, showing an improvement of the signal-to-noise ratio (SNR) with respect to previous analogue devices, and allowing acquisition of signals with sampling rate up to 20 Hz and source-detector distance up to 5 cm. A resting-state measurement on the motor cortex of a healthy volunteer was performed with an acquisition rate of 20 Hz at a 4 cm source-detector distance. The power spectrum for the cortical oxy- and deoxyhemoglobin is also provided.

## 1. Introduction

By exploiting picosecond pulsed lasers and single photon detectors, the time-domain (TD) near infrared spectroscopy (NIRS) technique allows retrieval of the absolute values of biological tissues’ optical properties, i.e., absorption (μ_a_) and reduced scattering (μ_s_′) coefficients. The acquired photon distribution of time-of-flight (DTOF) can be time-gated in order to better discriminate between the contribution of late photons, which traveled to a greater depth, and early photons, which traveled mostly through the more superficial layer [1]. Due to the poor signal-to-noise ratio (SNR), most of the TD NIRS instruments operate at an acquisition rate < 2 Hz, which is typically enough for monitoring the task-related cortical hemodynamic response that usually occurs with time constants of a few seconds [2]. However, for some specific applications, such as the monitoring of brain connectivity or resting-state oscillations, that sampling rate is too low. Spontaneous ongoing global activity of the brain at rest is highly structured in spatio-temporal patterns called resting-state networks. These fluctuations of brain activity exist even in the absence of tasks or stimuli [3], and were originally characterized by indirect and slow measurements of neuronal activity by blood oxygen level-dependent (BOLD) functional MRI (fMRI) thanks to the neurovascular coupling mechanism [4]. A non-invasive estimate of brain oscillations can also be achieved with the functional NIRS (fNIRS) technique, that exploits the different absorption spectra of oxygenated hemoglobin (O_2_Hb) and deoxygenated hemoglobin (HHb), as well as the penetration capability of NIR light in the human head [5]. By calculating the power spectral density of the signals related to the hemodynamic parameters in the frequency range <5 Hz, it is possible to study the presence of characteristic frequency peaks associated with physiological and/or pathological phenomena. Resting-state oscillation fNIRS studies were performed on patients with mild cognitive impairment [6], acute brain injuries [7] or autoregulation dysfunction [8]. By means of a multichannel setup, connectivity studies are also possible [9]. It is worth noting that all fNIRS studies were performed by a continuous wave (CW) or frequency domain (FD) approach, by which it was possible to reach a proper acquisition rate (e.g., 10 Hz) [10,11,12], differently from TD fNIRS.

During recent years, a huge development of the TD NIRS technique and instrumentation has been observed, with interesting advancements at the level of both research laboratories and companies; however, no sufficient SNR level was reached in order to increase the measurement acquisition rate [13]. It is worth noting that increasing the measurement SNR will not only allow faster acquisition rates, but also longer source-detector distance (*ρ*) measurements, as compared to the typical examples reported in the literature, i.e., <4 cm. Although the possibility to probe tissue in depth at null source-detector distance was also demonstrated by TD fNIRS, the use of larger *ρ* can be of help in overcoming non-idealities of the instrument response function (IRF) or in improving depth selectivity [14]. On the other hand, an increase in *ρ* corresponds to a decrease in signal at the detector (i.e., for an increase of 1 cm in the source-detector distance, we lose about one order of magnitude in the signal). The main technological bottlenecks from this point of view are: (i) the maximum average laser power is limited by safety regulations (<2 mW/mm^2^ for λ < 700 nm and from 2 up to 4 mW/mm^2^ for 700 < λ < 860 nm [15]); (ii) the need for high stability in time at the sub-nanosecond level, to avoid cross-talk between time drift and the estimation of optical properties; (iii) a limited signal harvesting efficiency of the detection line, i.e., the responsivity of the system. Koga et al. [16] attempted to develop a high power TD NIRS system by modifying an existing device. This instrument was employed in the assessment of superficial and deep muscle deoxygenation kinetics during heavy intensity exercises. They reported that it was possible to detect differences in optical properties up to 3 cm depth in a phantom, performing measurements at different source-detector distances (from 3 to 7 cm, at 1 cm steps in separate trials) but only at a 0.5 Hz acquisition rate. During the in vivo measurements (*ρ* = 3 and 6 cm), it was possible to find differences in superficial and deep muscle deoxyhemoglobin kinetics following the onset of heavy intensity exercise. In a recent work, Jiang et al. [17] presented a TD fNIRS optical tomography system (NIROT Pioneer) based on supercontinuum laser sources and SPAD detectors reaching a sampling rate of 2.5 Hz, aimed to perform DOT acquisitions. In this case, a fiber optical switch alternatively selected 11 source positions. The probe geometry allowed them to obtain an FOV with 2.5 cm diameter (i.e., *ρ* < 3 cm). A commercial solution, named “Flow”, was presented recently by Kernel (Kernel, Los Angeles, CA, USA, https://www.kernel.com/ (accessed on 22 December 2022)). They showed a multichannel wearable headset that measures brain activity [18], which potentially allows measurements up to a 100 Hz acquisition rate in the single channel, but now limited to 7.1 Hz to avoid cross-talk effects among channels. They also reported measurements performed at a 6 cm source-detector distance, but with a limited count rate (~10^4^ counts/s).

For what concerns the possibility to perform brain resting-state oscillation studies with TD fNIRS, to the best of our knowledge, there are only two previous attempts reported in the literature. In 2004, there was a 12 Hz acquisition from Themelis et al. (three independent channels with *ρ* = 1, 2 and 3 cm) [19], where they reported the presence of the heartbeat in the 830 nm cortical signal, without presenting a spectrum. They also affirmed that the selection of a proper time delay in the detected signal could increase the sensitivity of the TD fNIRS system to contributions coming from the deeper brain regions. The second attempt was performed by Kacprzak et al. in 2019 [20]. They developed an instrument based on pulsed semiconductor lasers and time-correlated single photon counting electronics (TCSPC), step-index fibers with 400 μm diameter and an IRF with a full width at half maximum (FWHM) of 500 ps. They presented in vivo acquisitions of the brain resting-state oscillations for both healthy subjects and patients with severe neurovascular disorders, with a 10 Hz acquisition rate, *ρ* = 3 cm and two locations on the head. They evaluated light attenuation (which reflects the superficial variations) and variance (more related to the cerebral compartment) of the DTOFs performing an FFT analysis of their changes. They showed interesting results for what concerns the presence of peaks in the frequency spectrum of the attenuation but, unfortunately, the measurement duration was set to only 10 min for the patients, and the frequency peaks in the variance spectrum were buried by the noise, indicating an insufficient SNR during the acquisition. In addition, they did not provide the same analysis for the hemodynamic parameters.

In this paper, we present a TD fNIRS setup where high power laser sources, hybrid photomultiplier tubes and custom detection bundles made of plastic optical fibers are employed. The instrument SNR is drastically improved with respect to previous analogue instrumentation, allowing acquisitions with a rate of 20 Hz and with source-detector distances up to 5 cm, both in phantom and in vivo applications. A first preliminary frequency domain analysis of the hemodynamic signals derived from TD fNIRS measurements is also provided.

## 2. Instrument Description

**Figure 1:**
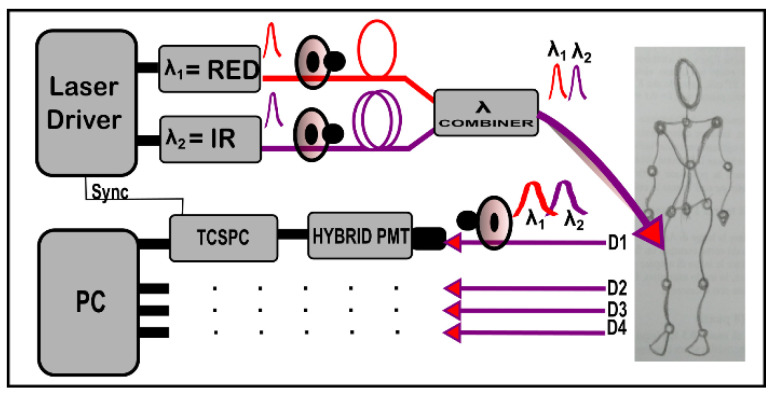
TD NIRS device scheme. λ = wavelength, PMT = Photomultiplier tube, TCSPC = Time-correlated single photon counting, D = Detection channel, Sync = Synchronization signal.

The TD fNIRS device is equipped with two high power pulsed diode lasers (LDH-P-C, Picoquant GmbH, Berlin, Germany) working respectively at 689.5 ± 0.5 nm (RED) and 828.5 ± 0.5 nm (IR). They are electronically driven at 80 MHz (PDL-828 Sepia II, Picoquant GmbH, Berlin, Germany) and emit pulses with a minimum pulse width of 72 ps (96 ps) for the RED (IR). The beam is coupled to step-index multimode glass optical fibers with a core/cladding diameter of 600/660 μm and NA = 0.22 (QMMJ-55-IRVIS-600/660-3-1.25, OZ Optics LTD., Ottawa, ON, Canada). It is possible to attenuate the beams by means of motorized and electronically driven continuous glass variable neutral density attenuators (NT43-770, Edmund Optics GmbH, Germany) inserted in a free beam region created by means of specific U-brackets (UB-12-11, OZ Optics LTD., Ottawa, ON, Canada). Before the sample, an optical beam combiner (FOBS-12P, OZ Optics LTD., Ottawa, ON, Canada) delays the IR wavelength and couples it with the RED one, in order to implement a time-multiplexing modality for the injection of light [21], i.e., both wavelengths interleaved in the same temporal window (12.5 ns) with a proper relative delay (6.4 ns). After the sample, diffused light is collected by means of four independent detection lines (D1–D4). Each detection line consists of: (i) a custom-made fiber optic bundle with 3 mm diameter and 1.25 m length, composed by 7 graded-index plastic optical fiber (POF) with NA = 0.3, core/cladding diameter of 900/1000 μm (FiberFin Inc., Yorkville, Illinois, USA) in hexagonal configuration; (ii) an attenuation stage provided by electronically driven continuous glass variable neutral density attenuators (NDC-50C-4-B, Thorlabs Inc., Newton, NJ, USA); (iii) a hybrid photomultiplier tube (PMA-50 Hybrid Series, Picoquant GmbH, Berlin, Germany). The DTOF acquisition is accomplished by a TCSPC unit (HydraHarp 400, Picoquant GmbH, Berlin, Germany) with short dead time (<80 ns), a maximum count rate per input channel of 12.5 × 10^6^ cps and an overall sustained throughput of about 40 × 10^6^ events/s, as summed over all channels. The whole system is controlled by a series of home-made units based on microcontrollers (DSPIC, Microchip Technology Inc., Chandler, AZ, USA), which also give an independent time basis and allow the synchronization with external hardware. In Figure 1, a scheme of the instrument is presented. The device was built with a modular structure, and it is equipped with a set of custom-made 3D printed probes made of a compatible material for diffuse optics applications [22]. The state-of-the-art instrument has a 1 × 4 configuration, i.e., one injection and four detection channels working in parallel.

## 3. Characterization Protocols

In this section, different international standardized characterization protocols employed for assessing the performances of diffuse optics instruments are presented. We also present the assessment of the maximum allowed count rate and acquisition rate. In addition, an in vivo measurement on an arm muscle is presented to validate the use of the device on humans. In the following sections, the optical parameters are estimated by a non-linear fitting procedure based on the Levenberg-Marquardt algorithm that minimizes the error (chi-square) between the measured DTOF and a theoretical function obtained by the convolution between the IRF and the analytical solution of the diffusion equation in a semi-infinite homogeneous medium [23].

### 3.1. Basic Instrumental Performance (BIP)

**Figure 2:**
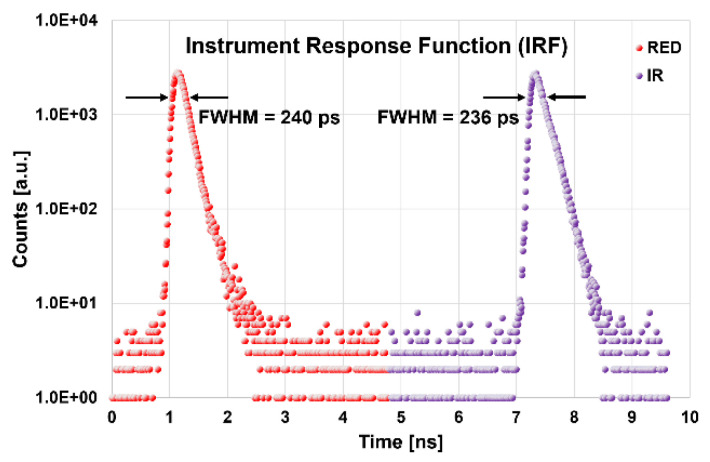
Typical acquisition window during an instrument response function (IRF) measurement. RED = 689 nm, IR = 828 nm, FWHM = Full width at half maximum, semi-logarithmic scale.

In the BIP protocol, the basic characteristics of the instrument are explored [24]. The maximum power exiting from the injection fibers towards the tissue is 1.90 mW (7.9 mW) for the RED (IR). These power settings were chosen in order to obtain an IRF with a FWHM of 240 ± 11 ps and 236 ± 12 ps, respectively, for RED and IR, expressed as the average ± standard deviation among the four detection lines. The width at 1% of the peak was 920 ± 40 ps (1020 ± 60 ps) for the RED (IR). In the same way, we can express the average responsivity S_avg_(λ), obtaining: S_avg_(RED) = (2.8 ± 1.4) × 10^−8^ m^2^sr and S_avg_(IR) = (1.5 ± 0.7) × 10^−8^ m^2^sr. The average afterpulsing ratio R_ap_(λ) is: R_ap_(RED) = 1.2 ± 0.8% and R_ap_(IR) = 1.0 ± 0.8%. The detector differential non-linearity is: ɛ_DNL_ = 5.9 ± 0.4%. The system requires a warm-up time of 110 (40) min in order to reach stability within ±1% (3%) of the final average values (counts, barycenter and FWHM of the IRF) calculated over the last 30 min of a 5 h acquisition. All the described parameters reflect those of previous TD fNIRS devices [25,26,27]. It is relevant that the setup characteristics, and in particular the choice of the proper optical fibers, allowed us to obtain IRFs with narrow FWHM, without undesired peaks due to internal reflections, as shown in Figure 2, providing the best conditions for a good fitting of the acquired data with the theoretical model [28].

### 3.2. Assessment of the Maximum Count Rate and Acquisition Rate

The upper limit to the maximum count rate is set by the detection and acquisition chain (see Section 2). The employed hybrid photomultipliers have a recommended upper limit in terms of count rate of 10^7^ counts/s (i.e., above this value, an electronic-controlled shutter automatically closes to prevent damage to the active area). The TCSPC system HydraHarp400, based on time-tagged time-resolved (TTTR) mode, guarantees a maximum count rate per input channel of 12.5 × 10^6^ cps and an overall sustained throughput of about 4 × 10^7^ events/second, as summed over all channels. On the other side, during the acquisition, the count rate is typically kept limited in order to operate in the single photon counting regime: it is necessary to guarantee that the count rate remains below 5% of the pulse rate, i.e., 4 MHz, since our lasers are working at 80 MHz, in order to avoid the “pile-up” effect [29]. Otherwise, the TCSPC system would register more than one photon per excitation cycle, causing a distortion of the DTOF and an error in the retrieval of the optical properties of the media under study. Recently, it was demonstrated, both with simulations and phantom acquisitions, that it is possible to work above the single photon statistics limit [30]. On these bases, we performed specific acquisitions in order to assess the maximum allowed count rate.

**Figure 3:**
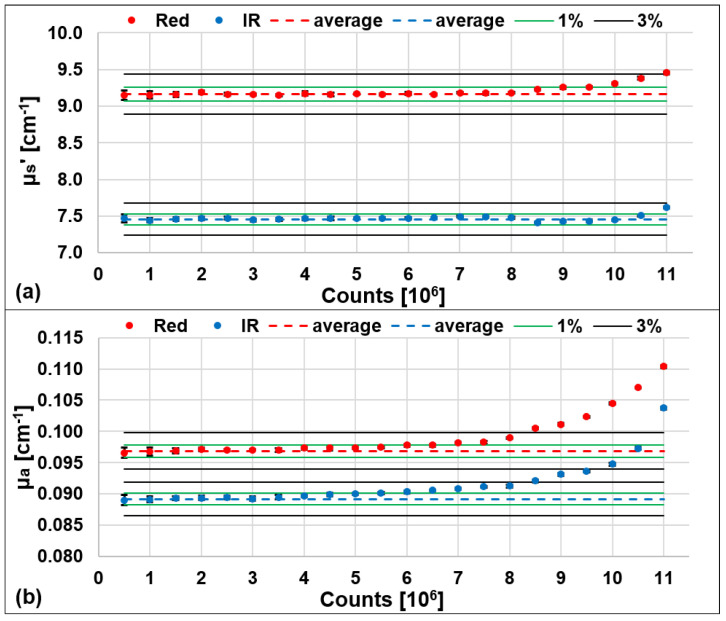
Averaged reduced scattering coefficient (μ_s_′, (**a**)) and absorption coefficient (μ_a_, (**b**)) over 10 repetitions and relative error bars, for different count rates. Red: RED wavelength, blue: IR wavelength. The dashed lines are the average μ_s_′ and μ_a_ retrieved for counts from 0.5 to 4 × 10^6^. The green and black lines represent the 1% and 3% error regions, respectively.

We then performed 10 repeated measurements, each with 1 s acquisition time and *ρ* = 3 cm, on a solid homogenous phantom (μ_a_ = 0.1 cm^−1^ and μ_s_′ = 10 cm^−1^ nominal optical properties) at different acquisition count rates: from 5 × 10^5^ ph/s up to 1.1 × 10^7^ ph/s on the board, with 5 × 10^5^ steps. The retrieved μ_s_′, shown in Figure 3a, showed small variations with the increasing of the number of acquired photons with <3% error with respect to the average calculated among the acquisitions at lower counts (from 0.5 to 4 × 10^6^) where the pile-up effect is negligible. For μ_a_ (Figure 3b), we observed an increasing trend with the increasing of the acquired counts, for both wavelengths. In order for the error not to exceed 3% with respect to its average value, calculated as for μ_s_′, it is necessary to set the injected photon count rate to a maximum of about 8 × 10^6^ ph/s, on the board, or equivalently 4 × 10^6^ ph/s for each wavelength with the relative DTOFs interleaved in the same temporal window.

Thanks to the previous findings and to the availability of a high number of detectable photons, we also tested the possibility to increase the acquisition rate, while maintaining enough detected photons to guarantee an optimal retrieval of the optical properties. We performed 10 repeated acquisitions for each acquisition time, on the same phantom as before with *ρ* = 3 cm. The acquisition sampling times were set to: 1, 0.1, 0.05, 0.01, 0.005, 0.004, 0.003, 0.002 and 0.001 s. We repeated the measurements at two different count rates: 2 × 10^6^ counts/s and 7 × 10^6^ counts/s per board.

**Figure 4:**
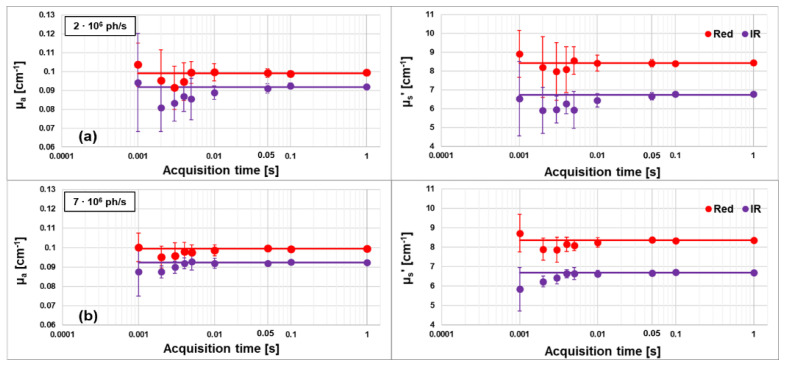
Absorption (μ_a_) and reduced scattering (μ_s_′) coefficients for both wavelengths as function of the acquisition time. Initial count rate set at 2 × 10^6^ counts/s (**a**) or 7 × 10^6^ counts/s (**b**). The horizontal lines represent the average value over the acquisitions at 1, 0.1 and 0.05 s.

In Figure 4, the retrieved values for μ_a_ (first column) and μ_s_′ (second column) at the two wavelengths are shown as functions of the acquisition time, when the initial count rate is set to 2 *∙* 10^6^ counts/s (first row) or 7 *∙* 10^6^ counts/s (second row). The solid horizontal lines represent the average value over the acquisitions at 1, 0.1 and 0.05 s. It is evident that, when reducing the acquisition time, the optical properties are obtained with a larger deviation from the average values and a greater dispersion (i.e., standard deviation).

It is then possible to estimate the minimum number of photons in the acquired DTOFs which guarantees a sufficient SNR for a reliable estimation of the optical parameters. For this purpose, we calculated the percentage coefficient of variation (CV%), defined as the standard deviation of a quantity divided by its average value and multiplied by 100 [31]. To obtain a CV < 1% for both optical coefficients and both wavelengths, a count rate of around 1.6 × 10^5^ counts/s for each acquisition, i.e., for each board, is necessary. That is equivalent, when wavelengths are interleaved, to 8.0 × 10^4^ counts/s for each wavelength. To guarantee enough photons, as stated from the CV parameter, we can use a minimum acquisition time of 0.1 s (0.03 s) with a count rate of 2 × 10^6^/s (7 × 10^6^/s).

### 3.3. Further Characterizations

The reproducibility (i.e., the capability to reproduce consistent values for the optical properties of the same phantom among four different days) and the linearity (i.e., the capability to correctly estimate the linear change in the optical properties) of our instrument were tested according to the MEDPHOT protocol [31].

We found that μ_a_ and μ_s_′ values showed variations lower than 3% around their average values calculated among the different days, showing an excellent reproducibility.

The linearity was tested on a set of 32 solid homogenous phantoms labeled with numbers from 1 to 8 and letters from A to D, in order to represent the different μ_a_ and μ_s_′ values, respectively (nominal optical properties from 0.01 to 0.49 cm^−1^ in 0.07 cm^−1^ steps for the absorption coefficient, and from 5 to 20 cm^−1^ in 5 cm^−1^ steps for reduced scattering coefficient, at 660 nm). We performed 10 repeated measurements, each with 1 s acquisition time, in reflectance geometry with *ρ* = 3 cm and a number of counts in the DTOF sufficient to guarantee a CV < 1% (see Section 3.2). Linearity was tested for both coefficients and both wavelengths by a linear interpolation. The R^2^ coefficients obtained were always >0.95, showing an excellent linearity, as shown in Figure S1 and Tables S1 and S2 of the Supplementary Materials.

**Figure 5:**
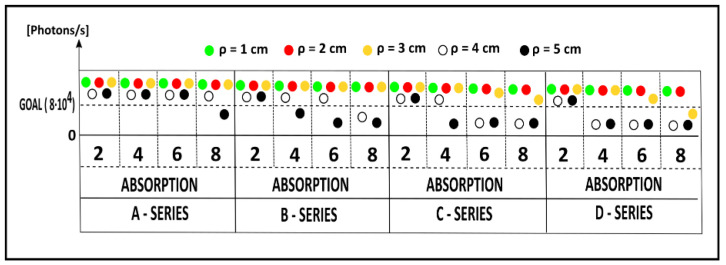
Number of photons/s for the RED wavelength, collected on solid phantoms for different values of absorption (labels 2, 4, 6 and 8: 0.07, 0. 21, 0.35, 0,49 cm^−1^, respectively) and reduced scattering (series A, B, C and D: 5, 10, 15 and 20 cm^−1^, respectively) with different source-detector distances *ρ* (different colors). In the figure, the count rate necessary to obtain a CV < 1% is shown as well.

Thanks to the increased SNR, we also investigated the possibility to perform acquisitions with different source-detector distances *ρ* (from 1 to 5 cm, at 1 cm steps) on the previous set of phantoms. During each measurement, we set the highest reachable count rate. In Figure 5, we show these count rates, for the RED, for all phantoms and source-detector distances (different colors). In this figure, we indicate the phantoms with their labels, and we set a horizontal line representing the value on the y-axis for the goal in terms of counts/s (8 × 10^4^ counts/s) necessary to obtain a CV < 1% (see Section 3.2). We can notice that, for the less scattering phantoms (A) it is always possible to reach enough counts, except for the most absorbent (8) for *ρ* = 5 cm (black dot). For the less absorbent phantoms (2), it is always possible to reach the goal counts, increasing the scattering (A–D) or the *ρ*. Moving towards more scattering and absorbing media, the measurement at *ρ* ≥ 4 cm is no longer achievable. Similar results were obtained for the IR wavelength, which shows in general a higher number of counts achievable, as shown during the BIP protocol. These data underline the improvement in terms of SNR of this TD fNIRS device over the previous ones published [21,27,32], with which it was typically not possible to measure phantoms D6 or D8 at *ρ* = 2 or 3 cm.

### 3.4. In Vivo Characterization Protocol: Arm Muscle Arterial Occlusion

In this section, we present an in vivo protocol to understand the feasibility of measurements on human tissues with a high acquisition rate (20 Hz) and long source-detector distances (up to 5 cm).

**Figure 6:**
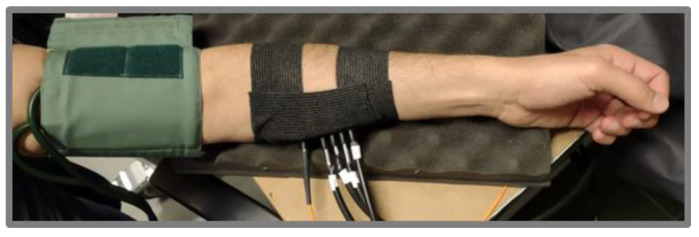
Probe placement during the in vivo occlusion on the arm muscle.

An arterial cuff occlusion (250 mmHg) of the left arm of a healthy adult volunteer was performed. The probe was placed on the internal side of the forearm (Figure 6), along the muscle fibers. The acquisition rate was set to 20 Hz and the measurements were performed, simultaneously, at 4 source-detector distances: from 2 to 5 cm, at 1 cm steps. The protocol consisted of 120 s baseline, 180 s occlusion and 300 s recovery. We noticed that, according to the results obtained in Section 3.2, the signal was sufficient to perform reliable acquisitions at 20 Hz at all interfiber distances. The absolute values for μ_a_ and μ_s_′ were obtained as explained in Section 3 for each acquisition point. The Lambert-Beer law was applied to estimate the O_2_Hb and HHb concentration at each time point during the experiment. A moving average of order 20 was applied to the retrieved hemodynamic parameters.

**Figure 7:**
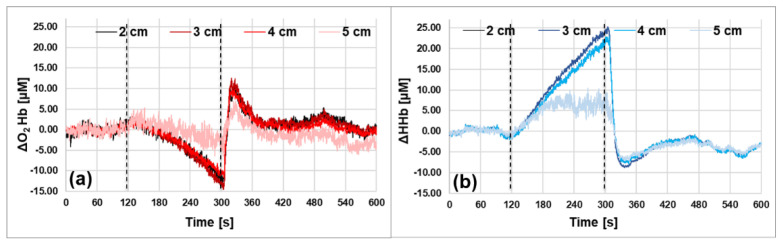
Hemodynamic parameters during an arterial arm occlusion. The dashed vertical lines indicate the start and the end of the occlusion period. The different lines represent the different source-detector distances (from 2 to 5 cm). (**a**) Oxyhemoglobin (O_2_Hb). (**b**) Deoxyhemoglobin (HHb).

In Figure 7, the time courses of the relative variations obtained for O_2_Hb and HHb are shown for all *ρ*-distances. The variations refer to the baseline values, calculated by averaging the concentration values found in the first 120 s of the experiment. As expected, during the occlusion the O_2_Hb decreases, since both veins and arteries are occluded, and no other oxygenated blood can enter in the investigated region. Conversely, the HHb increases because the muscle oxidative metabolism continues during the occlusion period. After the release of the cuff, we can observe the typical hyperemic peak. The qualitative behavior of the time courses at all *ρ*-distances is the same, but for *ρ* = 5 cm the amplitude of the variations is smaller. This behavior is more pronounced for the HHb. We do not have a clear explanation for this phenomenon. At first glance, it cannot be due to possible measurement faults, such as a lack of photons, because the SNR was sufficient at all source-detector distances. A possible explanation may be the heterogeneity of the tissue sampled at different *ρ*. This hypothesis was partially confirmed by an ultrasound exam of that arm region, which showed that tissue composition was different above and below a depth of 2.3 cm.

This preliminary measurement also demonstrates the feasibility of the application of the 20 Hz acquisition rate during in vivo measurements, with the possibility to follow big changes in absorption, such as the ones that occur during an arterial occlusion in the muscle. Changes around 15 μM for O_2_Hb and 25 μM for HHb were, in fact, detectable.

## 4. Cortical Resting-State Oscillations: Results and Discussion

In this section, we show an in vivo measurement with 20 Hz acquisition rate on the brain motor cortex of a healthy volunteer during a resting-state period and the resulting power spectrum for cortical O_2_Hb and HHb. This pilot study does not aim to explain the physiological origin of the peaks found in the frequency spectrum, but to demonstrate, for the first time, that it is possible to detect them by TD fNIRS.

We performed an acquisition on an adult healthy subject (male, 53 years old), in correspondence with the primary motor cortex area (C3 position according to the 10/20 EEG international system [33]). The subject relaxed in the supine position, with eyes closed, for 5 min. The acquisition rate was set to 20 Hz and the source-detector distance to 3 cm. The previous custom probe was placed on the scalp with a black auto-adhesive bandage, guaranteeing a good adhesion and avoiding ambient light leakage. The count rate of the measurement allowed performance of the acquisitions at 20 Hz, according to the results obtained in Section 3.2.

**Figure 8:**
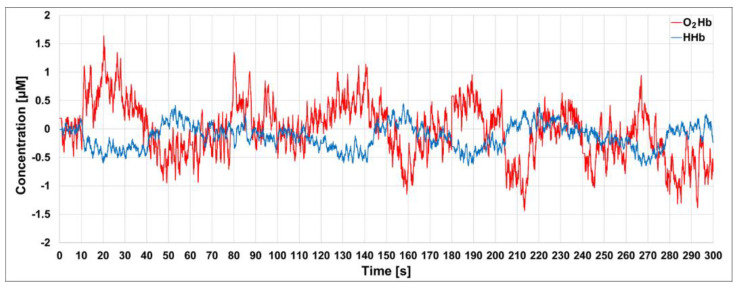
Time courses of the concentration of the cortical O_2_Hb and HHb, after subtraction of the average over the initial 5 s.

In order to enhance the contribution of the photons coming from deeper regions (late photons) from those coming from the more superficial regions (early photons), we modeled the tissue as a two-layer medium (up layer, UP; down layer, DW) and we calculated the time-dependent mean photon pathlengths in the UP and DW layers as described in Zucchelli et al. [34]. These pathlengths were used to estimate the absolute values of the cortical O_2_Hb and HHb hemoglobin concentrations, assuming a thickness of the upper layer of 1 cm (i.e., an equivalent thickness of the extra-cerebral tissue). For cortical O_2_Hb and HHb, we found an average of 44.71 μM and 17.94 μM, respectively, calculated over the initial 5 s. In Figure 8, the time courses of the concentration of O_2_Hb (red) and HHb (blue), after subtraction of the average values, are shown. A moving average of order 20 was applied to the retrieved hemodynamic parameters as well. As we can notice in Figure 8, a 1 s periodicity is clearly visible, superimposed on faster oscillations, for both hemoglobin species; this amplitude variability is higher for O_2_Hb than for HHb [10].

**Figure 9:**
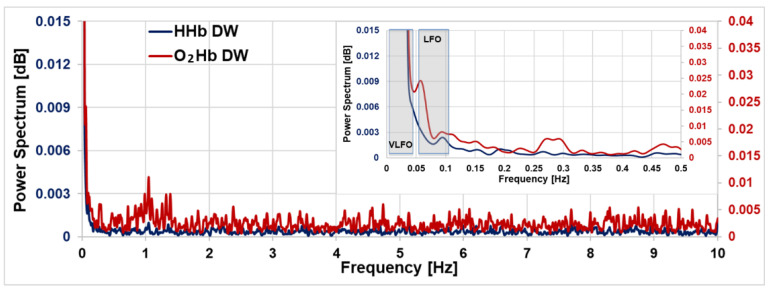
Power spectrum of the cortical O_2_Hb (red) and HHb (blue) hemoglobin for 5 min’s resting-state acquisition on the motor cortex. In the inset, a zoomed-in view of the frequencies ≤ 0.5 Hz is shown. LFO: Low frequency oscillation; VLFO: Very low frequency oscillation.

We then calculated the power spectrum for the cortical O_2_Hb and HHb with a custom-made code, based on the FFT algorithm (MATLAB 2021b, The MathWorks Inc., Natick, MA, USA), as shown in Figure 9. No filters were applied on the signal. At first, we can observe that the power spectrum amplitude is higher for O_2_Hb with respect to HHb, as previously shown in the literature with CW fNIRS [10]. In both spectra, it is possible to see the typical peak of the cardiac activity (~1 Hz), more pronounced in O_2_Hb as compared with HHb. Obrig et al. [10] have shown that the heartbeat produces changes in pressure, which are more visible in O_2_Hb, stating that this parameter should be more sensitive to systemic variations. In the O_2_Hb spectrum, a peak compatible with the respiration activity during rest (~0.2–0.3 Hz) can be recognized as well. In the HHb signal, a similar peak is present as well, but it is less evident. In addition, in previous CW-NIRS studies, the respiration peak could not always be visible [35].

In the figure inset, the power spectra for frequencies ≤ 0.5 Hz are shown. This frequency range is of particular interest since it includes the low frequency oscillations (LFOs, around 0.1 Hz) and the very low frequency oscillations (VLFOs, around 0.04 Hz) [10].

Firstly, we note that both in the O_2_Hb and in the HHb spectra a peak around 0.1 Hz is present, related to the intrinsic myogenic activity of the vascular smooth cell. As stated by Yücel et al. [36], at this frequency two different effects may be superimposed: the Mayer waves and vasomotion-flowmotion waves. The former are defined as waves in the arterial blood pressure, which cause an oscillation more visible in the superficial O_2_Hb [36]. Mayer waves should not be visible in the HHb [37]. On the contrary, vasomotion is defined as the oscillation in the blood vessels’ tone, which causes the cross-section of the blood vessel to oscillate, giving rise to the flowmotion [38]. This oscillation should be visible both in the cortical O_2_Hb and HHb, since in general the LFO amplitude should increase with the decreasing of the vessel diameter and the vessel diameter should decrease with the increasing of the depth (from the scalp to the cortex) [39]. The possibility to simultaneously quantify both hemoglobin species by TD fNIRS helps us in affirming that the peak at 0.1 Hz of the O_2_Hb could consist in a superposition of the two effects, i.e., Mayer waves and vasomotion-flowmotion, while the one found for HHb should be due to the vasomotion-flowmotion effect only.

If we now consider frequencies <0.1 Hz, in the O_2_Hb spectrum an oscillation around 0.06 Hz is clearly visible, possibly related to the neurogenic activity of the vessel walls. To better understand its origin, further experiments are required, where some physiological changes can be induced to observe the respective changes in the spectra. Of course, the concurrent acquisition of the main physiological parameters (such as heartbeat, respiratory rate, arterial blood pressure, blood volume pulses and others) can help in a better interpretation of the whole spectrum.

Finally, in Figure 9, it is possible to notice a strong frequency component at less than 0.04 Hz, for both hemoglobin species. This range covers the neurogenic activity of the vessel wall and the vascular endothelium function. In a future work, in order to remove the continuous component from the frequency spectrum and thus recover the characteristic peaks in this region, a detrending algorithm has to be applied. Furthermore, other methodologies should be used to obtain sharper peaks, more comparable with the previous literature findings, such as the power spectral density (PSD) estimate via Welch’s method.

## 5. Conclusions

In this paper, we presented a TD fNIRS device reaching a higher SNR as compared with previous similar instruments, obtained by combining more powerful lasers and a more efficient detection system. As shown in Section 3.2 and Section 3.3, it was possible to collect enough signal at the 20 Hz acquisition rate to reliably (CV < 1%) retrieve the optical properties of homogeneous phantoms with high absorption (0.35 cm^−1^) and highly reduced scattering (20 cm^−1^) coefficients at a 3 cm source-detector distance. Measurements with a source-detector distance up to 5 cm were also achievable on homogeneous phantoms mimicking the optical properties of a biological medium (μ_a_ = 0.1 cm^−1^ and μ_s_′ =10 cm^−1^). In general, we demonstrated the possibility to perform measurements with an up to 5 cm interfiber distance in reflectance geometry, at a maximum acquisition rate of 20 Hz on diffusive samples with optical properties like those of biological tissues. This result, to the best of our knowledge, has never been reached by any other TD fNIRS instrument to date. Thanks to the four independent detection lines, it was possible to perform acquisitions in parallel in four different acquisition points. In Section 3.4, we showed the possibility to employ this device during in vivo measurements on the arm muscle, thus retrieving the absolute values of the hemodynamic parameters, employing source-detector distances up to 5 cm.

Furthermore, in Section 4, we showed the power spectra for the absolute values of both cortical O_2_Hb and HHb obtained by a TD fNIRS acquisition. This preliminary acquisition, on a healthy subject, aimed to prove the feasibility of performing measurements on the cerebral cortex with high sampling rate (20 Hz) by TD fNIRS, rather than explain in depth each resulting spectral peak; to the best of our knowledge, this result has never been achieved to date, as already underlined in the Introduction. In particular, thanks to this acquisition we showed that by TD fNIRS: (1) it is possible to detect the intracranial heartbeat signal, in particular in the cortical O_2_Hb signal; (2) it is possible to observe the intracranial respiration, at least in the O_2_Hb signal, that was observed by Kacprazak et al. [20] in the superficial layer only; (3) we increased the SNR, obtaining a non-noisy spectrum by acquisitions of only 5 min. In the only previously published paper, they needed longer measurements (20 min) and affirmed that, for the patients, 10 min of acquisition at 10 Hz were not sufficient, and that some interesting frequencies were buried under the noise; (4) we were able to provide the spectra of the cerebral O_2_Hb and HHb, starting from their absolute values, by one measurement at a single source-detector distance. Thanks to this opportunity, we were able to distinguish important spectral contributions in the frequency range below 0.5 Hz.

We think that this study opens up the possibility to perform TD fNIRS measurements at a high acquisition rate (up to 20 Hz), filling the gap with CW fNIRS instruments and other previous techniques such as fMRI. In particular, if only an exploration of the more superficial layer of the brain cortex with fNIRS is possible, there are a series of advantages in choosing this optical technique. It is possible to perform acquisitions at the bedside and to guarantee a continuous monitoring. fNIRS is less sensitive to motion artifacts and the signal does not present physiological noise due to respiratory and cardiac activities, which cause an unwanted modulation in fMRI signal [40,41]. In addition, if the fMRI signal carries information only about the BOLD, with fNIRS and in particular TD fNIRS, it is possible to decouple the contributions of the oxygenated and deoxygenated blood. In this way, the capability of this technique to provide a more accurate estimation of cortical hemodynamic parameters can also be fully exploited in cerebral resting-state oscillation studies and, in the future, by increasing the measurement points, in brain connectivity studies as well.

Of course, further work is necessary to understand the best analysis method for the extrapolation of the hemodynamics frequency spectra. Theoretical simulations will also be necessary to define constraints, if any, about the length of the experiment, the number of photons needed to distinguish two different peaks and other technical aspects. Furthermore, it will be necessary to employ additional physiological sensors, in order to acquire at least heartbeat and respiration rate, and to increase the number of subjects involved, for a better interpretation of the in vivo results.

## Data Availability

Data underlying the results presented in this paper are not publicly available at this time but may be obtained from the corresponding authors upon reasonable request.

## Supplementary Materials

The following supporting information can be downloaded at: https://www.mdpi.com/article/10.3390/s23010196/s1, Figure S1: Linearity plots according to MEDPHOT protocol; Table S1: Linear interpolation goodness for absorption coefficient; Table S2: Linear interpolation goodness for scattering coefficient.
