# Supplementary material for: Reliable Fast (20 Hz) Acquisition Rate by a TD fNIRS Device: Brain Resting-State Oscillation Studies
Source: Sensors (Basel). 2022 Dec 24;23(1):196. doi: 10.3390/s23010196 (PMC9823873; doi:10.3390/s23010196)
Supplement: Supplementary file 1 [file sensors-23-00196-s001.zip › sensors-2070267-supplementary.pdf]

Supplementary materials

Figure S1. “Linearity plots according to MEDPHOT protocol”

The linearity was tested on a set of 32 solid homogenous phantoms labelled with numbers from 1 to 8 and letters from A to D, in order to represent the different μ_a and μ_s' values, respectively (nominal optical properties from 0.01 to 0.49 cm^{-1}, in 0.07 cm^{-1} steps for the absorption coefficient and from 5 to 20 cm^{-1}, in 5 cm^{-1} steps for reduced scattering coefficient, at 660 nm) [29]. We performed 10 repeated measurements, 1 s each, in a reflectance geometry with ρ = 3 cm and a DTOF count number enough to guarantee a CV < 1% (see Sec. 3.2). Linearity was tested for both coefficients and both wavelengths with a linear interpolation. The R^2 coefficients obtained were always > 0.95 showing an excellent linearity, as shown in Figure S1, Table T1 and T2. Same results were obtained for the IR wavelength.

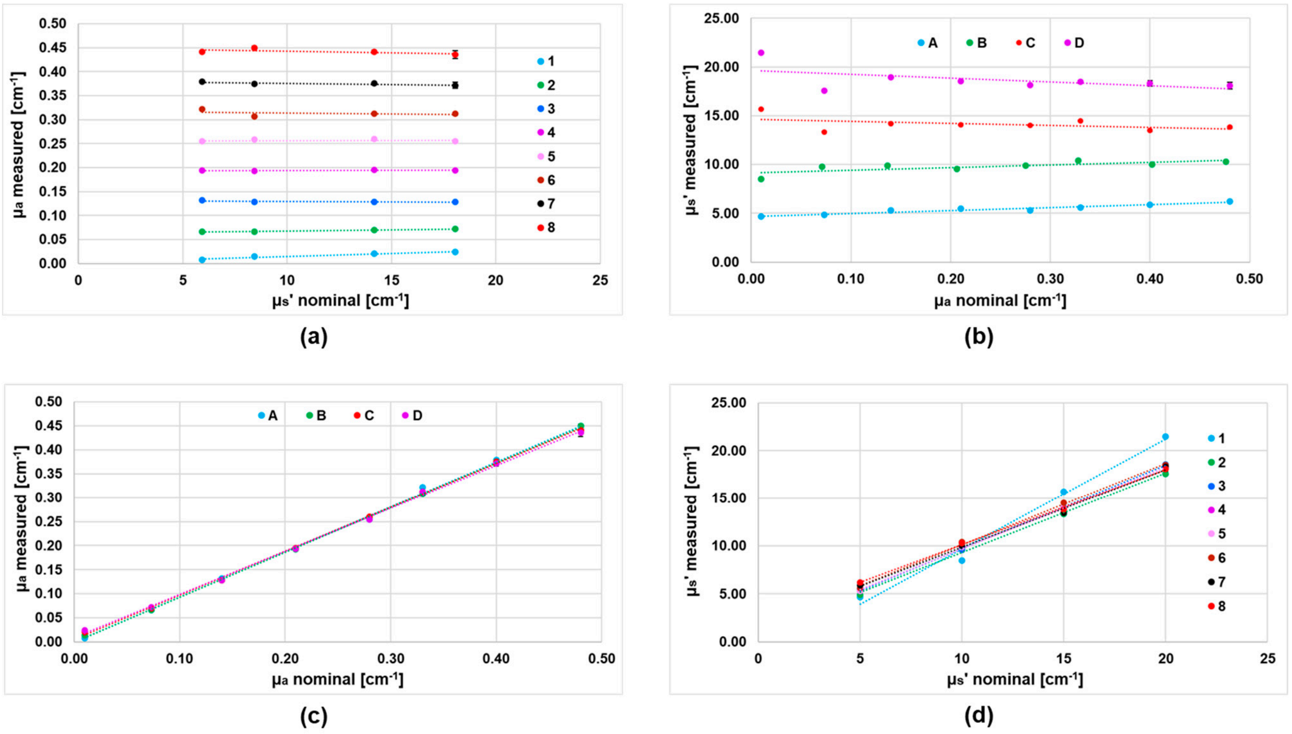

Figure S1. Linearity for RED wavelength (690 nm). The points represent the retrieved optical coefficients averaged over the 10 repetitions, and the error bars represent the standard deviation. The dotted lines are obtained as the linear interpolation of each data series. Letters and numbers represent the identification labels of the phantoms.

Table S1. "Linear interpolation goodness for absorption coefficient"

Table S1. R^2 coefficients obtained with the linear interpolation of $\mu_{a\_measured}$ vs $\mu_{a\_nominal}$ at RED wavelength. Results are averaged over phantoms with same nominal scattering.

$R^2(\mu_{a_{measured}}\ vs\ \mu_{a\_nominal})$			
**A**	**B**	**C**	**D**
0.9982	0.9982	0.9992	0.9939

Table S2. "Linear interpolation goodness for scattering coefficient"

Table S2. R^2 coefficients obtained with the linear interpolation of $\mu'_{s\_measured}$ vs $\mu'_{s\_nominal}$ at RED wavelength. Results are averaged over phantoms with same nominal absorption

$R^2(\mu'_{s_{measured}}\ vs\ \mu'_{s\_nominal})$							
**1**	**2**	**3**	**4**	**5**	**6**	**7**	**8**
0.9976	0.9962	0.9997	0.9995	0.9994	0.9978	0.9962	0.9987

.
